# The threat of colistin resistance among carbapenem-resistant *Klebsiella pneumoniae* isolates in Iran

**Published:** 2018-04

**Authors:** Mehri Haeili, Mohammad Mehdi Feizabadi

**Affiliations:** 1 Department of Biology, Faculty of Natural Sciences, University of Tabriz, Tabriz, Iran; 2 Department of Microbiology, School of Medicine, Tehran University of Medical Sciences, Tehran, Iran

Colistin belongs to polymyxin family of antibiotics which are polypeptide bactericidal agents acting mainly by disruption of outer-membrane in Gram-negative bacteria (GNB). This antibiotic group (including polymyxin B and colistin) was approved for clinical use in the late 1950s but fell out of favour soon because of the reported high incidence of nephrotoxicity. With increasing the emergence of extensively-drug resistant (XDR)-GNB and paucity of new marketed antibiotics, polymyxins have recently regained significant clinical interest. They are currently considered as the last-line defense against problematic XDR-GNB notably carbapenem-resistant *Enterobacteriaceae* (CRE) and *Acinetobacter baumannii*. Colistin revival in clinical settings has increased the inevitable risk of emerging resistance. Resistance to colistin in GNB is known to be mainly mediated by chemical modification or complete loss of the antibiotic target, LPS. Recently the transmissible plasmid mediated resistance mechanism has been identified in *Enterobacteriaceae* posing a significant threat to infection control programs. Despite having a very similar chemical structure and indistinguishable antimicrobial activity *in vitro*, colistin and polymyxin B (PMB) differ mainly in the form administered parenterally. While PMB is administered directly as its active form, colistin is formulated as an inactive prodrug, colistin methanesulfonate (CMS) which itself lacks antibacterial activity and requires to be converted *in vivo* to colistin in a reaction which occurs slowly and incompletely. As a consequence, plasma concentrations of colistin rise slowly resulting in lower plasma concentrations in renally competent patients. Indeed, it is estimated that only <20%–25% of a dose of CMS is converted to active colistin *in vivo* (1). This is associated with reduced bacterial killing and may facilitate selection of colistin resistant mutants (1, 2). PMB on the other hand has superior clinical pharmacological properties over the CMS due to its rapid attainment of target concentrations, and may be a better therapeutic option for treatment of bloodstream infections (BSIs) (1, 3). PMB and CMS also differ in the way they are eliminated with the first is being mainly cleared by nonrenal pathway and the latter is eliminated predominantly by kidneys. Therefore, urinary concentrations of colistin exceed those that can be achieved with PMB, highlighting CMS as a better option for the treatment of urinary tract infections compared to PMB (1, 3). Different countries vary in their access to parenteral formulation of CMS or PMB. In Iran, clinicians use CMS parenteral formulation for treatment of serious infections caused by XDR bacterial isolates most commonly *A. baumannii* and *Klebsiella pneumoniae*. Recently, we have encountered with outbreaks of different clones of colistin resistant carbapenemase-producing *K. pneumoniae* (CR-KP) isolates at one of the largest hospitals of the country, Iran. The CR-KP stains were found to be isolated from different clinical samples including blood, tracheal aspirate, urine and wound and most of the patients from which CR-KP isolates were obtained characterized with BSIs. Assessing the molecular mechanisms of colistin resistance in the *K. pneumoniae* isolates showed that resistance was related to chromosomal mediated mechanisms but not plasmid encoded *mcr* genes. Indeed, genetic alterations in the *mgrB* gene including nonsense mutations and insertion of IS elements were found to be the major mechanisms mediating colistin resistance in these isolates (4). MgrB, a small transmembrane protein with 47 amino acids acts as the negative regulator of PhoP/PhoQ, a two-component regulatory system that regulates the expression of genes involved in LPS modifications and colistin resistance. Increased consumption of CMS in the hospital for treatment of infections caused by carbapenemase producing isolates of *K. pneumoniae* and also *A. baumannii* has probably provided the selective pressure for development of resistance to this agent. We have also previously demonstrated that repeated/long term colistin exposure is a great risk factor for emergence of colistin resistant mutants among *A. baumannii* isolates (5). Due to a high prevalence of CR-KP isolates in BSIs, it is hypothesized that administration of PMB for treatment of bloodstream infections may offer some benefits. Moreover, it has been described that conditions such as suboptimal dosing or prolonged monotherapy, are significantly associated with advent of colistin resistance. Therefore, clinicians should consider combination therapies containing polymyxins rather than colistin monotherapy for treatment of CRE infections. This approach improves clinical outcomes in critically ill patients and suggests favorable option for minimizing the emergence of polymyxin resistance. Likewise, tigecycline-based combination regimens containing colistin or other antibiotics for treatment of infections caused by carbapenem-resistant *K. pneumoniae* have shown promising outcomes (6). Isolation of patients colonized or infected with colistin resistant strains are important for preventing patient-to-patient transmission in the hospital environment. Clinical microbiologists, infectious diseases specialists and clinical pharmacists should collaborate closely for a successful management of antimicrobial resistance at hospitals. Clinical microbiologists should actively participate in antimicrobial steward-ship committees and providing therapy advice. However, the participation of clinical microbiologists in such programs is limited in some clinical settings and distancing of microbiology laboratories will make the situation even more complicated. At this moment, while we await the development of novel antibiotics with activity against the difficult-to-treat Gram-negative ‘superbugs’, we must also follow rational approaches to optimize the use and efficacy of older antibiotics including polymyxins. Also, more studies are required about the clinical pharmacokinetics/pharmacodynamics of polymyxins notably PMB, and factors that influence the activity of polymyxins, and development of resistance.
